# Structural Transitions in Nanosized Zn_0.97_Al_0.03_O Powders under High Pressure Analyzed by in Situ Angle-Dispersive X-ray Diffraction

**DOI:** 10.3390/ma9070561

**Published:** 2016-07-12

**Authors:** Chih-Ming Lin, Hsin-Tzu Liu, Shi-Yao Zhong, Chia-Hung Hsu, Yi-Te Chiu, Ming-Fong Tai, Jenh-Yih Juang, Yu-Chun Chuang, Yen-Fa Liao

**Affiliations:** 1 Department of Applied Science, National Hsinchu University of Education, Hsinchu 30014, Taiwan; g9925056@mail.nhcue.edu.tw (S.-Y.Z.); g10025051@mail.nhcue.edu.tw (C.-H.H.); vinnychiu0109@gmail.com (Y.-T.C.); 2 Department of Chemical Engineering, National Tsing Hua University, Hsinchu 30013, Taiwan; missliu29@yahoo.com.tw; 3 Chemical Systems Research Division, Chung-Shan Institute of Science & Technology, Taoyuan 32546, Taiwan; 4 Department of Physics, National Tsing Hua University, Hsinchu 30013, Taiwan; 5 Department of Electrophysics, National Chiao Tung University, Hsinchu 33013, Taiwan; 6 National Synchrotron Radiation Research Center, 101 Hsin-Ann Road, Hsinchu Science Park, Hsinchu 30076, Taiwan; chuang.yc@nsrrc.org.tw (Y.-C.C.); liao.yenfa@nsrrc.org.tw (Y.-F.L.)

**Keywords:** aluminum-doped zinc oxide, zinc-blende, phase transition, angle-dispersive X-ray diffraction

## Abstract

Nanosized aluminum-doped zinc oxide Zn_1−*x*_Al*_x_*O (AZO) powders (AZO-NPs) with *x* = 0.01, 0.03, 0.06, 0.09 and 0.11 were synthesized by chemical precipitation method. The thermogravimetric analysis (TGA) indicated that the precursors were converted to oxides from hydroxides near 250 °C, which were then heated to 500 °C for subsequent thermal processes to obtain preliminary powders. The obtained preliminary powders were then calcined at 500 °C for three hours. The structure and morphology of the products were measured and characterized by angle-dispersive X-ray diffraction (ADXRD) and scanning electron microscopy (SEM). ADXRD results showed that AZO-NPs with Al content less than 11% exhibited würtzite zinc oxide structure and there was no other impurity phase in the AZO-NPs, suggesting substitutional doping of Al on Zn sites. The Zn_0.97_Al_0.03_O powders (A_3_ZO-NPs) with grain size of about 21.4 nm were used for high-pressure measurements. The in situ ADXRD measurements revealed that, for loading run, the pressure-induced würtzite (B4)-to-rocksalt (B1) structural phase transition began at 9.0(1) GPa. Compared to the predicted phase-transition pressure of ~12.7 GPa for pristine ZnO nanocrystals of similar grain size (~21.4 nm), the transition pressure for the present A_3_ZO-NPs exhibited a reduction of ~3.7 GPa. The significant reduction in phase-transition pressure is attributed to the effects of highly selective site occupation, namely Zn^2+^ and Al^3+^, were mainly found in tetrahedral and octahedral sites, respectively.

## 1. Introduction

With the large exciton binding energy (~60 meV) and wide direct band gap (~3.37 eV) [1], zinc oxide (ZnO), a II–VI semiconductor, has been considered as one of the most prominent candidates that can realize the applications of futuristic high brightness light sources operating in the ultraviolet (UV) region. Besides pursuing the high-purity bulk, it is also important to obtain the material in various forms of nanostructures, such as nanoparticles, nanorods and nanwires. There have been many different methods being used for synthesis of nanostructures such as laser ablation for nanoparticles [2,3], chemical vapor deposition for nanowire [4,5], thermal evaporation on pulsed laser deposition for nanowire [6], atomic layer deposition for nanowires and vertically aligned nanorods [7]. Furthermore, it has been demonstrated that, by alloying with MgO [8] and CdO [9,10], the importance of ZnO is further enhanced from the viewpoint of band gap engineering as well as forming p−n junctions for laser diode or light emitting diodes. On the other hand, aluminum is one of the metal ions widely used in doping ZnO to modify its electrical and optical properties [11,12]. Since the exact amount of *x* in the obtained Zn_1−*x*_M*_x_*O (M = Ga^3+^, In^3+^, Al^3+^, etc.) is playing a pivotal role in determining the ultimate electrical and optical properties, researchers have long been searching for methods that can provide more accurate estimation on solubility limit of metal ions in ZnO [13,14,15,16]. Shirouzu et al. [14] and Serier et al. [17] pointed out that substitution of Al^3+^ for Zn^2+^ can be extremely difficult because of the differences in lattice constants, ionic radius, oxidation state, and the preferred coordinates. In particular, it has been demonstrated that Zn^2+^ and Al^3+^ are preferably occupying the tetrahedral and octahedral sites in ZnO system, respectively [18]. Thus far, extending the solubility limit of Al^3+^ in ZnO still remains a major challenge and extensive efforts have been put forth to develop more efficient and reliable synthesis processes for raising the doping level of Al^3+^. Chen et al. [19] reported that different synthesis methods might lead to the different Al^3+^ solubility limits in ZnO systems. For example, for chemical synthesis routes, the Al solubility limits in ZnO were 10 mol %, 4 mol %, and 0.3 mol % by alkali precipitation [20], the facile solvothermal route [19], and Pechini route [17], respectively. Mohanta et al. [4] indicated that Al doping is very effective in reducing the oxygen vacancies, especially when deposited at low oxygen ambient pressure during chemical vapor deposition, leading to a suppression of the broad visible emission band and enhanced UV emission in photoluminescence spectra. Recently, it has been demonstrated that Al-doped ZnO (AZO) can exhibit satisfactory electrical and optical properties for transparent conducting applications [21,22], thus being able to serve as a competitive alternative to indium tin oxide (ITO), the most commonly used transparent conducting oxide (TCO) material. Moreover, compared with the rarity and expensiveness of indium, ZnO and Al are relatively abundant, cheap and non-toxic, making it even more appealing for replacing ITO with AZO [23]. In this study, we introduce a chemical precipitation method with urea addition to obtain AZO nanoparticles with even higher Al solubility limit.

Moreover, recent reports indicated that the phase transition pressure, *P*_tr-nano_, of ZnO nanocrystals is very much size dependent. Namely, for grain size of 10, 12, 18, 30 and 65 nm *P*_tr-nano_ varied monotonically from 16.6 [24], 15.1 [25], 12.0 [26], 10.8 [27], to 10.0 GPa [27], respectively. Bayarjargal et al. [24] suggested that the variation of *P*_tr-nano_ could be fitted by an empirical linear expression: *P*_tr-nano_(*D*) = 10.1 + 56.13 × (1/*D*), where *D* is the average grain size of the ZnO particles. This was due to the higher surface energy of the B1 phase relative to the B4 phase in the ZnO system. For the ZnO system, the transition pressure shifted to higher values in nanocrystals with decreasing crystal size [24]. In contrast to ZnO system, the AlN nanocrystals had lower transition pressures than bulk samples, which has been attributed to the higher surface energy of the B4 phase relative to the B1 phase [24]. Subsequently, it was shown that doping could further reduce the phase transition pressure of ZnO [28,29]. For example, in Mn-doped ZnO, the starting (completing) pressures of the B4-to-B1 phase transition were changed from 9.5(11) GPa for pure ZnO to 8.3(12) GPa for Zn_0.99_Mn_0.01_O [28] and 6.5(9) [28] or 7.35(12.46) GPa [29] for Zn_0.98_Mn_0.02_O crystals, respectively. Regardless of the relatively scattered variations in the completing pressure, the existence of manganese ions in the ZnO crystal appears to have substantially reduced the phase transition barrier, and hence lowered the starting pressure required to trigger the phase transition. In this respect, due to the highly selective site occupancy preference of Al^3+^ in ZnO, it should be interesting to delineate the effect of aluminum doping on the path of B4-to-B1 phase transition, which has remained largely unexplored. In this respect, the AZO-NPs with *x* = 0.03 (A_3_ZO-NPs) synthesized in air by the present chemical precipitation method were used to investigate the pressure-induced phase transition, compressibility, equation of state and phase transition paths by angle-dispersive X-ray diffraction (ADXRD) to unveil the effect of the doped Al^3+^ ions on Zn-O bonding.

## 2. Experimental Details

AZO-NPs with a würtzite type hexagonal structure were prepared by chemical precipitation [30] using zinc nitrate (Zn(NO_3_)_2_·6H_2_O), aluminium nitrate (Al(NO_3_)_3_·9H_2_O) and urea (CO(NH_2_)_2_) as the starting materials. Thermogravimetric (TG) measurements were conducted with a thermobalance (TGA Q50, New Castle, DE, USA) in nitrogen atmosphere (purity 99.995%) with a heating rate of 10 °C/min up to 1000 °C. The XRD data for nanosized aluminum-doped zinc oxide Zn_1−*x*_Al*_x_*O, *x* = 0.01, 0.03, 0.06, 0.09 and 0.11 under ambient conditions were collected by a Rigaku Rotaflex (Tokyo, Japan) 18-kW rotating anode diffractometer with graphite monochromatized Cu-Kα radiation and a scanning step of 0.02° in the 2θ range 25°–75°.

For high-pressure measurements, the A_3_ZO-NPs was ground into powder in a zirconium oxide ball mill with acetone for 2 h. After grinding, the powder was kept at room temperature for two weeks to release the possible residual stress resulting from the grinding process. The obtained A_3_ZO-NPs was loaded into a symmetric diamond anvil cell (DAC). The initial thickness of the 301 stainless steel gasket was 250 μm, which was then pre-indented to a thickness of about 70–80 μm. A sample chamber of about 235 μm in diameter was drilled at the center of the indented gasket using a discharge machine. Fine (1–2 μm) ruby powders were simultaneously placed inside the sample chamber with a pressure-transmitting medium (PTM), consisting of a mixture of methanol and ethanol with a ratio (in volume) of 4:1 (methanol/ethanol). The ground samples were placed on top of the ruby powders and the ruby fluorescence spectra were measured at the same spot. The pressures for high-pressure ADXRD measurements within the DAC were determined by the shifts of fluorescence lines of ruby, using the calibration of Mao et al. [31]. In particular, Angel et al. [32] and Klotz et al. [33] have addressed the issue of nonhydrostaticity arising from silicone oil medium. In this respect, Klotz et al. [33] further pointed out that the 4:1 methanol–ethanol mixture was the most commonly used PTM and had been investigated by a number of groups. All of them conclusively showed that the glass transition of the PTM was at 10.5 GPa and the effect of deuteration occurred at 10.5 ± 0.5 GPa. The aim of our measurements is not only to investigate the effects of the doped aluminum element on beginning pressure of the structural phase transition in ZnO but also to look at the mechanism of phase transition. Hence, the 4:1 methanol-ethanol mixture seems to be a suitable PTM in the present high-pressure study.

ADXRD measurements were performed using the beamline BL12B1 at SPring8 (Hyogo, Japan) and the wavelength was 0.6191 Å (20.0273 keV). Final values of the lattice parameters for samples with different Al doping concentrations and high-pressure measurements for A_3_ZO-NPs were obtained using Rietveld refinement, which refined user-selected parameters by minimizing the difference between an experimental pattern and a shape based on the composite crystal structure and instrumental parameters. A program (General Structure Analysis System, GSAS) [34] with an editor (graphical user interface, GUI, EXPUGI, Santa Fe, NM, USA) was applied to control the progress of the Rietveld-type fit in obtaining unit cell parameters, volumes and *u* parameters. There are many parameters such as scale factor, background, lattice parameters, zero shift (specimen displacement), phase, constraints, and thermal parameters to be adopted in refining the information obtained in this work. With Al atoms substituted for zinc atoms, the same fractional coordinates, the same U_iso_ in Zn and Al atoms and the occupancy of 0.95 and 0.03 for Zn and Al, respectively, are applied in Rietveld-type fitting processes. Theoretically, *n* diffraction peaks, where each diffraction peak acts as an observation, can refine n-1 parameters. The grain size of the A_3_ZO-NPs was calculated using the Williamson–Hall equation reported previously by Mote et al. [35]. GSAS also offers a constant wavelength (CW) X-ray profile function, LX, which allows us to obtain the information of particle size via the following expression:

particle size = (18000 × K × λ)/(π × LX),(1)

where K is the Scherrer constant (typically ~0.9) and the unit for particle size is the same as that of the wavelength (λ); both are in Angstroms (Å). The morphology and composition of the A_3_ZO-NPs were examined with a thermal-type field-emission scanning electron microscope (FESEM, JSM-7000F, Tokyo, Japan) with a detection limit of 0.01 wt % and with an inductively coupled plasma with atomic emission spectroscopy (ICP-AES, ICP Optima 2100DV, Markham, ON, Canada), respectively. From the energy-dispersive spectrometer (EDS) and ICP-AES analyses, the obtained Al doping concentration in the present A_3_ZO-NPs was verified to be consistent with that introduced during the synthesis processes.

## 3. Results and Discussion

**Figure 1:**
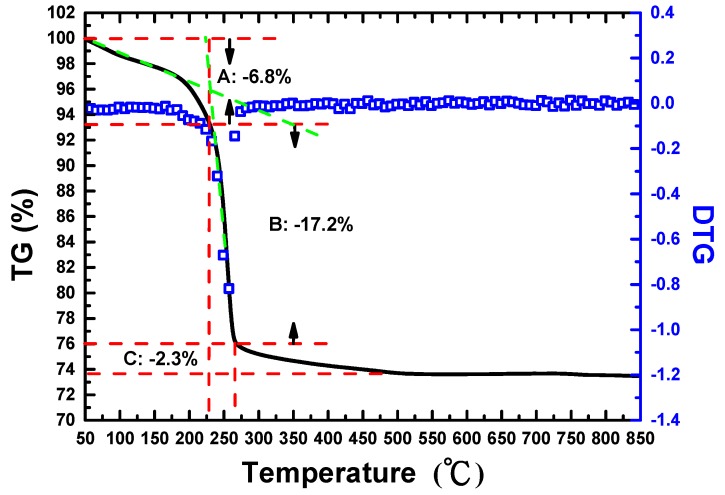
Thermo-gravimetric (TG) and differential TG (DTG) measurements for the precursor of AZO-NPs.

Figure 1 shows the TG and derivative thermo-gravimetric (DTG) plots to illustrate the reaction and crystalline conditions of the AZO-NPs precursor obtained from the chemical precipitation synthesis processes. It can be seen that a continuous loss of mass (~6.8 mass %) occurs within the 50 to 225 °C temperature range (range A), accounting for the desorption processes of free water, physically adsorbed water and organic reagents. A relatively precipitous mass loss (~17.2 mass %) taking place in a much narrower temperature range from 225 to 270 °C (range B) indicates that it might involve the overlapping of decomposition, viz. dehydration, and decomposition, viz. oxidation. According to the chemical reactions: Zn(OH)_2_ → ZnO + H_2_O and 2Al(OH)_3_ → Al_2_O_3_ + 3H_2_O and the molar mass of Zn(OH)_2_, ZnO, Al(OH)_3_ and Al_2_O_3_, which are 99.38, 81.38, 78 and 102 g/mol, respectively, the theoretical weight loss percentage can be calculated as following: [(99.38 − 81.38)/99.38 × 100] × 0.97 + [(78.2 − 102)/(78.2) × 100] × 0.03 = 18.61%. After taking into account the effect from range A (the first stage of removing the adsorbed water), the weight loss of single range B segment can be calculated from Figure 1, giving rise to: (93% − 76%)/93% = 18.28%, close to the theoretical value indicated above. Figure 1 also shows a total residual value of 80 mass % in the TG plot and, more clearly in its DTG plot, with the major signal at 250 °C being corresponding to dehydration. An additional continuous mass loss of ~2.3 mass % seen in the temperature range of 270 to 500 °C (range C) indicates the nitrate decomposition. For temperatures higher than 500 °C, the TG plot displays a plateau and a stable product for the crystalline precursor of AZO-NPs. Based on the TG and DTG-plots analyses, we then performed the subsequent thermal processes and calcined the AZO-NPs precursors at 500 °C for three hours to obtain the AZO-NPs to be used in the present study.

**Figure 2:**
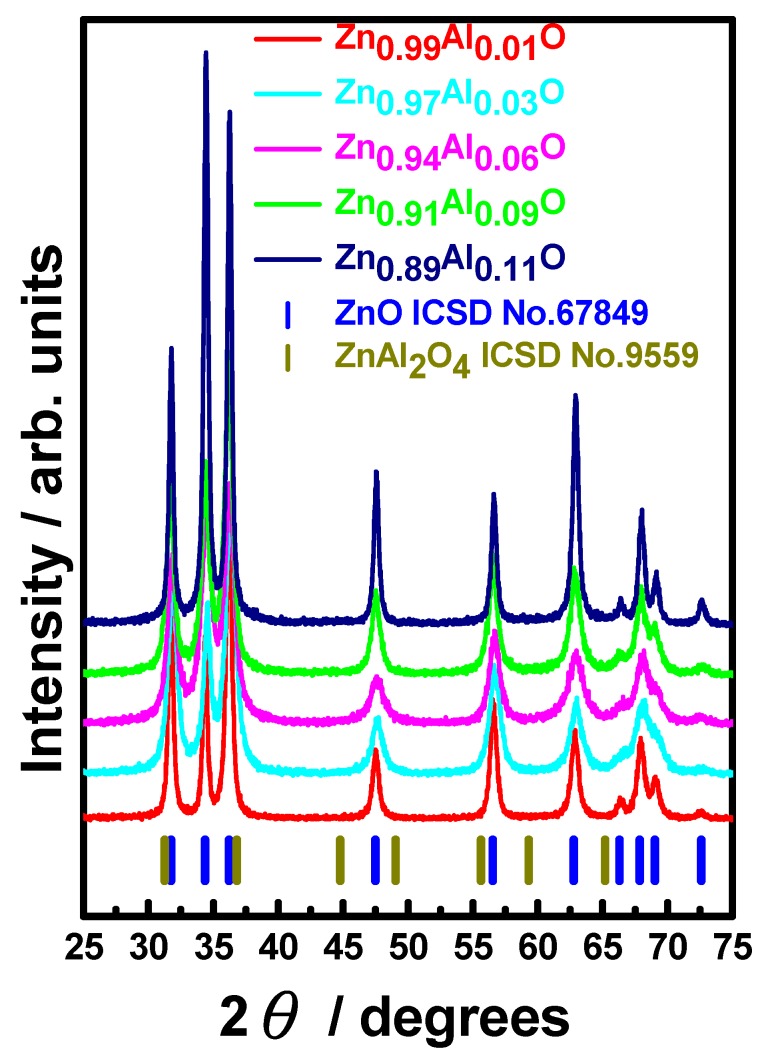
XRD patterns of AZO NPs with various Al concentrations. All the samples were prepared at 500 °C.

Figure 2 shows the ADXRD patterns for AZO-NPs with various Al doping concentrations obtained by the processes described above together with the ICSD index of ZnO (No. 67849) and ZnAl_2_O_4_ (No. 9559). From Figure 2, it is apparent that all AZO-NPs are attributable to the würtzite (B4) phase for hexagonal structure of ZnO. The unit-cell parameters at the ambient conditions for all AZO-NPs obtained by the present chemical precipitation process are listed in Table 1. There is an obvious trend of decreasing *c*-axis lattice constant with increasing nominal Al concentration, which is consistent with the observation reported by Brehm et al. [36]. Namely, larger Al doping concentration would lead to more reduction in unit cell volume. Since the ionic radii for Al^3+^ and Zn^2+^ are 67.5 and 88 pm [36], respectively, it is reasonable to expect the lattice reduction provided that Al has been doped in ZnO lattice substitutionally. The fact that there is no Al or oxide cluster, such as the ZnAl_2_O_4_ phase, being observed in the present AZO-NPs indicates that Al atoms might have doped into the ZnO lattice by substituting for zinc atoms. Moreover, the nearly linear dependence of lattice reduction as a function of nominal Al doping concentration further suggests that within the composition range presented in the present study, the linear Vegard’s law could be used to evaluate the Al doping concentration in the ZnO structure. To reinforce the above arguments, the EDS and ICP-AES analyses for AZO-NPs were conducted to offer an independent check. The results are listed in Table 2. From Table 2, it is evident that the concentrations of Al obtained by EDS are consistent with those obtained from ICP-AES analyses, except for Zn_0.99_Al_0.01_O, presumably due to the resolution limit of EDS analyses. In addition, by using an isotropic LX profile term in GSAS analyses, the average grain size of the present AZO-NPs are calculated to be about 52.1(1), 21.4(1), 18.0(1), 17.6(1), and 19.9(3) nm for Zn_0.99_Al_0.01_O, Zn_0.97_Al_0.03_O, Zn_0.94_Al_0.06_O, Zn_0.91_Al_0.09_O, and Zn_0.89_Al_0.11_O at ambient condition, respectively. In any case, the above XRD, EDS and ICP-AES observations seem to suggest that uniform doping of Al up to 11 at % is evidently achieved. Nevertheless, as had been pointed by Ku et al. [37], since the bonding energy of the Al-O bond (~511 kJ/mol) is much larger than that of Zn-O bond (~271 kJ/mol), one might reasonably assume that the doped Al can easily break the Zn-O bond and occupy the Zn site. Consequently, larger Al doping concentration would lead to more reduction in the grain size of AZO-NPs, which is qualitatively consistent with the grain size analyses described above, except for Zn_0.89_Al_0.11_O. In addition, since the ionic radii for Al^3+^ and Zn^2+^ are 67.5 and 88 pm [36], respectively, one expects that the substitution of Al on Zn site will progressively reduce the lattice parameters with increasing Al doping concentration, which is also consistent with the results shown in Table 1. It is noted, however, as the concentration of Al reaches 11 at %, the grain size appears to be slightly increased and becomes larger than that of samples with 6 and 9 at % Al doping concentrations. From both of the results of Al concentration dependence of grain size and lattice parameters, as well as the ICP-AES measurement results, it is suggestive that the maximum aluminum doping limit being achieved by the present method might fall in the neighborhood of 9 at %. Since our primary interest in high-pressure measurements is to unveil the effects of Al-doping on the phase transition of ZnO, we chose to focus on A_3_ZO-NPs because this concentration seems to be high enough to reveal the Al-doping effect on high-pressure phase transition of ZnO yet low enough to not introduce other complications. For A_3_ZO-NPs, the EDS results reveal that the concentration for O, Al, and Zn are 49.98 at %, 1.51 at %, and 48.51 at %, respectively. The result confirms that the doping concentration Al (in replacing Zn) is indeed very much consistent with the nominal doping concentration, indicating the effectiveness of chemical precipitation method in obtaining a high doping concentration. It is also suggestive from the EDS and ICP-AES results shown in Table 2 that there might still be a small number of oxygen vacancies existing in the present A_3_ZO-NPs because the doping level of Al might still be not enough. We note that this, in fact, is in line with the observation by Mohanta et al. [4], where decrease of oxygen vacancies with increasing Al concentration has been evidently demonstrated by using optical spectroscopic techniques. However, since there is an inherent detecting limit for EDS, especially for light atomic species such as oxygen, further analyses are required to settle the issue.

**Figure 3:**
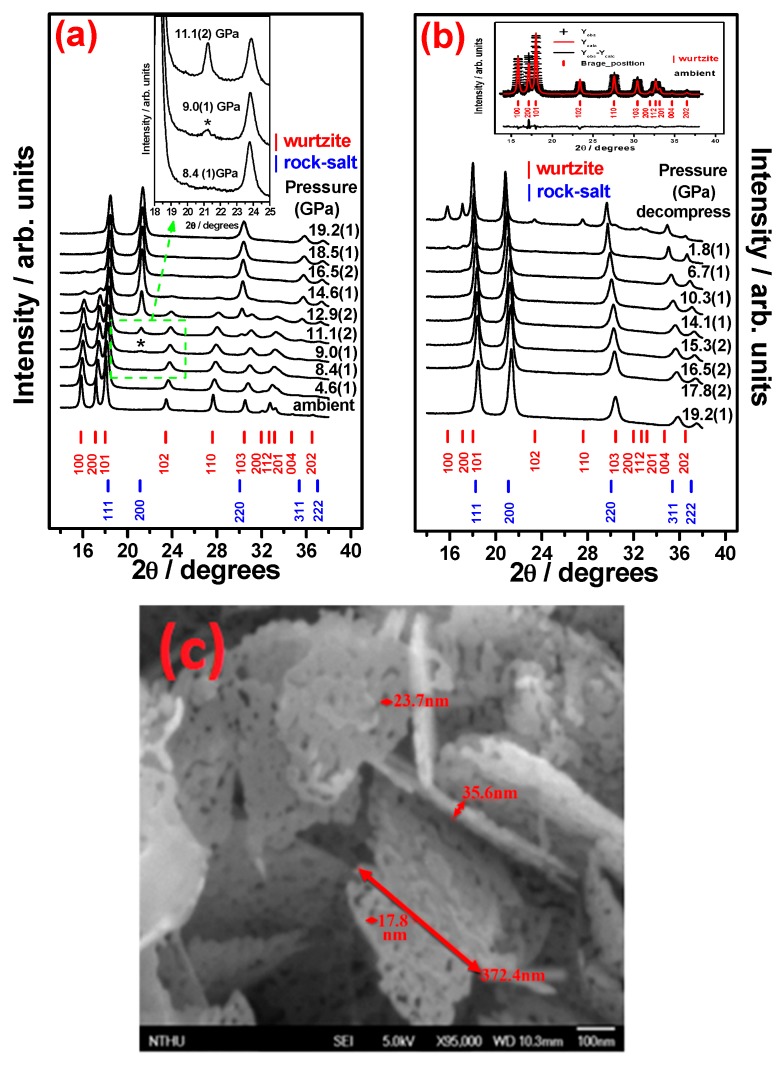
(**a**) Representative ADXRD patterns of bulk A_3_ZO-NPs at elevated pressures and decompressure. Bulk A_3_ZO-NPs exhibited a phase transition with an onset pressure of 9.0(1) GPa marked with * in the inset; (**b**) representative ADXRD patterns of bulk A_3_ZO-NPs at degraded pressures and decompressure. The black daggers represent experimental points and the red solid line represents Rietveld refined data and the bottom line shows the difference between the experimental and refined data and the marked 2θ positions are the allowed Bragg peaks in the inset; (**c**) SEM image of A_3_ZO-NPs and more detail shows the hollowed plate-like and granular microstructures.

In situ ADXRD spectra of the obtained A_3_ZO-NPs measured with a loading run up to 19.2(1) GPa and unloading run to ambient pressure are shown in Figure 3a,b, respectively. We first look at the pattern obtained at ambient pressure prior to run-up loading. It evidently reveals that the obtained A_3_ZO-NPs are attributable to the hexagonal structured würtzite (B4) phase. The GSAS analysis indicates that the obtained ADXRD pattern contains 11 independent reflections. From the ambient pressure results displayed in Figure 3a, the unit-cell parameters of the AZO-NPs are: *a* = *b* = 3.247(1) Å, *c* = 5.199(1) Å, and V/Z (a.k.a. V_o_) = 23.737(2) Å^3^. These values are very close to values of *a* = *b* = 3.247 Å, *c* = 5.201 Å reported by Farbod et al. [38]. Nevertheless, they are slightly smaller than the reported value of *a* = *b* = 3.254(1) Å, *c* = 5.210(1) Å and V/Z = 23.885 Å^3^ (Inorganic Crystal Structure Database (ICSD) No. 67849), *a* = *b* = 3.254 Å, *c* = 5.200 Å by Suwanboon et al. [20], and *a* = *b* = 3.251 Å, *c* = 5.209 Å by Chen et al. [19] for hexagonal structured pristine ZnO. Moreover, as can be seen from Figure 3a (similar to those displayed in Figure 2), all ADXRD patterns of AZO-NPs show that there is no discernible diffraction peak of impurity phases, such as Al or oxide clusters of Al, like the ZnAl_2_O_4_ phase, indicating that Al^3+^ ions are doped into the ZnO lattice by substituting for Zn^2+^ ions as indicated by Farbod et al. [38]. In our XRD results for AZO nanoparticles, it can be seen that the profiles for observed and calculated ones are matching perfectly to each other and all the experimental peaks agree well with the allowed Bragg diffractions for würtzite structure, as shown in the inset of Figure 3b. The Rietveld refinement converged to w_Rp_ = 2.16%, Rp = 1.10% and χ^2^ = 0.36 for the ADXRD pattern at ambient pressure. The inset of Figure 3b evidently displays a very good fit between the observed and calculated patterns. The calculated theoretical density of 5.691(1) g∙cm^−3^ in ambient condition is also slightly larger than the value of 5.656(1) g∙cm^−3^ listed in the ICSD No. 67849. The slightly reduced unit-cell parameters for the A_3_ZO-NPs, thus, may have resulted from the introduction of the dilute Al^3+^ ions randomly substituting Zn^2+^ ions. Serier et al. [17] reported that there were two doping mechanisms for Al-doped ZnO. One is the replacement of Zn^2+^ cation by an Al^3+^ cation paired with a free electron: ZnO + Al → Al_Zn_O + Zn + e^−^, where Al_Zn_O denotes replacing one Zn site with an Al ion. The other is replacing three Zn^2+^ cations by two Al^3+^ cations accompanied by a compensation cationic vacancy: 3ZnO + 2Al → 2Al_Zn_O + 3Zn + ▯_Zn_, where ▯_Zn_ denotes the compensate cationic vacancy. Since the ionic radii for Al^3+^ and Zn^2+^ are 0.675 and 0.880 Å, respectively [36], the uniform reduction (i.e., around 0.2%) of the unit-cell parameters revealed in our ADXRD results suggest that the Al-doping is rather homogeneous and is more likely following the former doping mechanism. AZO is a transparent conducting oxide (TCO) with reasonable electrical resistivity (~10^−4^ Ω cm) and large transmittance (~92%), making it an attractive candidate for transparent conducting electrode in photoelectric devices. It has been demonstrated that an accompanying free electron is paired with the process when a Zn^2+^ cation is replaced by an Al^3+^ cation, which is responsible for the increased conductivity [39,40,41]. Furthermore, Figure 3c shows the SEM image for the A_3_ZO-NPs obtained by the present chemical precipitation and 500 °C calcination processes. It reveals that the morphology of the A_3_ZO-NPs is primarily of hollowed plate-like and granular shape. The volume of the hollowed plate-like structures is estimated to be about 372.4 × 372.4 × 35.6 μm^3^. The calculated grain size of the A_3_ZO-NPs using the Williamson–Hall equation [34] is plotted with 4sin*θ* as the *x*-axis and ${\left(\beta^{2}-\beta_{ins}^{2}\right)}^{1/2}\cos\theta$ as the *y*-axis. By extrapolating the plot linearly, the intercept at the *y*-axis gives an estimated grain size of 22.5(1) nm for the present A_3_ZO-NPs. Alternatively, by using an isotropic LX profile term in GSAS analyses, the grain size of the present A_3_ZO-NPs is calculated to be about 21.4(1) nm at ambient condition, indicating that each hollowed plate-like (or granular) microstructure may contain numerous of AZO grains. Due to the requirements of accuracy in GSAS analyses, the grain size of A_3_ZO-NPs is taken to be about 21.4(1) nm in this work. In any case, the above observations indicate that the chemical precipitation method can influence the shape of the A_3_ZO-NPs substantially, albeit that uniform doping of Al is evidently achieved.

Next, we turn to the results of pressure-induced phase transitions in the present A_3_ZO-NPs. The inset of Figure 3a shows that, as the pressure reaches 9.0(1) GPa, a discernible new diffraction peak (marked with an asterisk) corresponding to the 200-reflection of B1 phase starts to emerge for A_3_ZO-NPs. Moreover, although the intensity of the new peak grows substantially with the increasing pressure, coexistence of diffraction peaks from the original B4 phase is evident over a wide range of pressures (9.0(1)~16.5(2) GPa), indicating that the phase transition is taking place locally rather than over the entire bulk. At 9.0(1) GPa, the lattice parameters are *a* = 3.207(1) Å, *c* = 5.094(1) Å and V/Z = 22.697(3) Å^3^ for the B4 phase and *a* = 4.228(1) Å, V/Z = 18.764(7) Å^3^ for the B1 phase, respectively, corresponding to an ~17.3% volume reduction. The calculated theoretical densities are 5.952(1) and 7.172(1) g∙cm^−3^ for B4 and B1 structures at 9.0(1) GPa, respectively, and both are increased with increasing applied pressure. It is noted that the theoretical density of B1 structure is denser than that of the B4 structure under high pressure. At 16.5(2) GPa, the lattice parameters are *a* = 3.190(1) Å, *c* = 5.079(1) Å and V/Z = 22.383(5) Å^3^ for the B4 phase and *a* = 4.189(1) Å, V/Z = 18.379(3) Å^3^ for the B1 phase, respectively. In this case, the corresponding volume reduction is ~16.9%, which is slightly smaller than that at 9.0 GPa, presumably due to the difference in pressure dependent bonding stiffness for the two phases. As mentioned above the doping mechanism for A_3_ZO-NPs is following the reaction of replacing a Zn^2+^ by an Al^3+^ paired with a free electron [17], and the Zn^2+^ and Al^3+^ are preferably occupying the tetrahedral and octahedral sites in ZnO system [18], which, in turn, is expected to result in the difference of bonding stiffness between the two phases. The onset pressure of the transition from B4 to B1 phase is 9.0(1) GPa, the reflections of the B4 phase of A_3_ZO-NPs disappeared completely and only reflections of B1 phase are observed for pressures above 18.5(1) GPa. Based on the thermodynamics arguments the transition pressure should be proximate, or equal, to the onset pressure. Therefore, in the present study the transition pressure (*P*_tr_) of B4 to B1 for A_3_ZO-NPs is taken as 9.0(1) GPa. In general, the starting and completing pressures of the B4-to-B1 phase transition for pure bulk ZnO probed by various methods were around 8.8–10.0 GPa and 8.8–15.0 GPa, respectively [24,42,43,44,45,46,47,48]. The onset pressure obtained in the present study is within the reported range of starting phase transition for ZnO under high pressure. The experimental total enthalpy change (Δ°*H*) for the B4 to B1 structure transformation at 9.0(1) GPa may be estimated as follows [49]: Δ°*H* ≈ *P*_tr_ (−Δ*V*) = 9.0 GPa × 3.933 Å^3^ × 6.02 × 10^23^/mole = 21.309(1) kJ∙mole^−1^, which provide enough internal energy for reconstructing the B1 structure. On the other hand, in an unloading process, as displayed in Figure 3b, the B1 phase of A_3_ZO-NPs is found to remain metastable until the pressure is ramped down to 1.8(1) GPa and a small amount of B4 phase starts to emerge slowly. When the pressure is relaxed back to the ambient pressure, a tremendous amount of strain is expected to be released in the sample and a substantial part of the A_3_ZO-NPs reverted to the B4 phase. Nevertheless, as is evident from the decompressed ADXRD pattern shown in Figure 3b, there is still a significant amount of the metastable B1 phase remaining in the sample. Even more interestingly, the corresponding diffraction peaks of the remaining B1 phase appear to remain very sharp without noticeable broadening. Desgreniers [45] and Decremps et al. [50] reported that the B4-to-B1 phase transition is completely reversible for bulk ZnO. However, the metastable B1 phase can still be detected in the recovered AZO-NPs as a mixture with the B4 phase in some cases, such as ZnO nanocrystals [25], ZnO nanowires [51], and ZnO nanobelts [52]. This is indicative that the pressure-induced phase transition path in the present A_3_ZO-NPs could be highly reversible.

**Figure 4:**
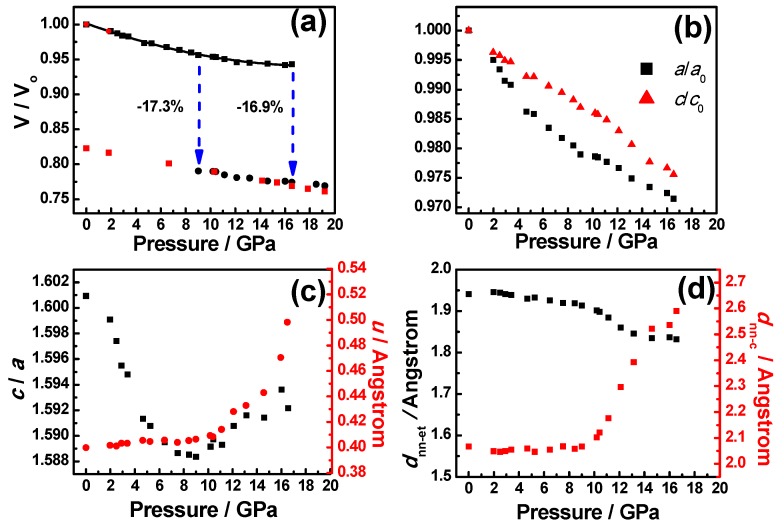
(**a**) Pressure dependence of the V/V_0_ of bulk A_3_ZO-NPs at 300 K. The black solid line is the fitting result using the third-order BM EoS for B4 phase; (**b**) the change of bulk A_3_ZO-NPs cell parameters *a*, and *c* with increasing pressures; (**c**) pressure dependence of *c*/*a* ratio (black solid squares) and the internal structural parameter *u* of würtzite structure A_3_ZO-NPs (**red** solid circles) as a function of pressure obtained from Rietveld refinement and (**d**) the pressure dependence of the bond length of equilateral triangle (*d*_et_) and the nearest-neighbor distance of O to Zn(Al) parallel to the *c*-axis (*d*_nn-c_).

Figure 4a shows the volume vs. pressure data measured at the ambient temperature for A_3_ZO-NPs. The solid black rectangular and solid black circular symbols in Figure 4a are representing the corresponding phases of B4 and B1 identified during the loading up run, and the solid red rectangles are representing the phases observed during the unloading process, respectively. The data for the B4 phase were fitted to the third-order Birch–Murnaghan equation of state (BM EoS) [49] as:

(2) P(V)=3B02[(V0V)73−(V0V)53]{1+34(B0′−4)[(V0V)23−1]}

where *V* and *V*_0_ represent the high-pressure and ambient-pressure unit cell volume, respectively. The black solid line in Figure 4a is the fitting result using the third-order BM EoS for B4 phase. The obtained values of the zero-pressure isothermal bulk moduli $\left(B_{0}\right)$ and its first derivative with respect to pressure $\left(B_{0}^{\prime }\right)$ for B4 phase are 146(7) GPa and 7(2), respectively. These values are slightly larger than the value reported by Liu et al. [46] in Table 3, suggesting that the structure may have been more susceptible to the applied external pressure. Nevertheless, it is noted that the PTM used could play an important role in pressure-induced phase transition of ZnO. Liu et al. [46] pointed out that the hydrostatic pressure conditions usually yields more meaningful bulk modulus while the nonhydrostatic or quasihydrostatic conditions tends to overestimate the magnitude of *B*_0_. Similar conclusion about the significant role played by the PTM has also been reached in pressure-induced phase transition of InAs [53]. The pressure evolution of the normalized lattice parameters *a/a*_0_, and *c*/*c*_0_ of the B4 phase are presented in Figure 4b. Around *P* = 8.4(1) GPa, the linear compressibility along the *c*-axis *K_c_* = −(1/*c*)(*dc*/*dP*)*_T_* = 3.078 × 10^−3^ GPa^−1^ is larger than that along the *a*-axis *K_a_* = 1.509 × 10^−3^ GPa^−1^. Unit-cell lattice parameters *a*, *c*, *c*/*a*, *u*, the nearest-neighbor distance between O^2−^ and Zn^2+^(Al^3+^) ions measured the *c*-axis (*d*_nn-c_) and the bond lengths of equilateral triangle (*d*_nn-et_) of A_3_ZO-NPs of B4 phase before 16.5(2) GPa are listed in Table 2. The fact that the compression rate along the ***c***-axis is higher indicates that the spacing between O^2−^ and Zn^2+^(Al^3+^) is more compressible than those between Zn^2+^(Al^3+^) and Zn^2+^(Al^3+^) ions. Consequently, the lattice is more susceptible to deform along the *c*-axis under pressure. In Figure 4c, the internal structural parameter *u* and the *c*/*a* ratio obtained from the Rietveld refinements for A_3_ZO-NPs are plotted as a function of pressure. Our data indicate that the *c*/*a* ratio is ~1.601(1) for B4 phase at ambient pressure, which is slightly smaller than the reported values of 1.61 for pure ZnO [54,55] and deviates substantially from the value of 1.633 for the ideal *c*/*a* ratio of würtzite structure with a hexagonal unit cell, respectively. Kisi et al. [56] pointed out that larger electronegativity difference between the two constituents in hexagonal compounds tends to result in more deviation of the *c*/*a* ratio from the ideal value. The electronegativity using the Pauling scale [57] for Al, Zn and O are 1.61, 1.65 and 3.44, respectively. The electronegativity difference between Al and O is slightly larger than that between Zn and O. Thus, the fact that the *c*/*a* ratio for A_3_ZO-NPs is smaller than that for pure ZnO is also in line with the conjecture that the doped aluminum is randomly distributed within the ZnO lattice. Moreover, as depicted in Figure 4c, the pressure dependence of the axial ratio *c*/*a* for the B4 phase exhibits a “turning point” at 9.0(1) GPa, coinciding with the onset of the B4-to-B1 phase transition. For pressures lower than this point, the *c*/*a* ratio follows an ordinary decreasing trend with increasing pressure. However, beyond this point the *c*/*a* ratio increases abruptly with further compression until the transformation is completed. We note that similar pressure-dependent behavior of *c/a* was observed previously [51]. The relationship between the *c*/*a* ratio and *P* can be fitted by a quadratic polynomial function with: *c*/*a* = 1.602(1) − 0.003(1) *P* + 0.001(1) *P*^2^. It is noted that, unlike that reported by Saitta et al. [54], our results do not show an abrupt drop in the *c*/*a* ratio to 1.29 amid the pressure-induced phase transition. On the other hand, in our case, the *u* value increases slightly from 0.400(1) to 0.405(1) as the pressure is increased from the ambient pressure to 8.4(1) GPa. Thereafter, the *u* values increase quickly up to ~0.498(1) at 16.5(2) GPa, beyond which the B4 phase converts to the B1 phase completely. Limpijumnong et al. [55] proposed a hexagonal path model to elucidate the pressure-induced B4-to-B1 phase transition. The model features an initial continuous decease in the *c*/*a* ratio accompanied by an increasing *u* value to 0.5. Then the γ angle opens up from 60° to 90° to trigger the formation of the B1 phase. In our data, the *c*/*a* ratio is observed to decrease monotonically with increasing pressure before the phase transition started. The obtained *u* value also increases continuously with increasing pressure and finally reaches a value of 0.498(1). These evidences are suggestive that the B4-to-B1 phase transformation in the A_3_ZO-NPs is more likely proceeding via the hexagonal path rather than the tetragonal one [54]. In fact, similar conclusions had been drawn for ZnO in other structural forms, such as nanocrystals [58], nanowires [51] and nanotubes [59]. It is suggestive that such a pressure-driving phase transition path might be a genuine intrinsic property of ZnO. Özgür et al. [60] reported that the *u* value is alternatively described as the bond length or the nearest-neighbor distance between O^2−^ and Zn^2+^(Al^3+^) ions measured the *c*-axis (*d*_nn-c_) divided by *c*. In Figure 4d, the bond length of equilateral triangle (*d*_nn-et_) and *d*_nn-c_ are plotted as a function of pressure. It is seen that *d*_nn-c_ remains almost constant ~2.067(1) Å at ambient pressure with a very minor decreases to ~2.058(1) Å at *P* = 8.4(1) GPa. Beyond *P* = 9.0(1) GPa, *d*_nn-c_ increases rapidly up to a value of 2.590(1) Å (corresponding to near 25% increase) at *P* = 16.5(2) GPa. On the other hand, *d*_nn-et_ decreases only slightly from 1.940(1) Å to 1.919(1) Å in the pressure range of ambient to 8.4(1) GPa. However, beyond *P* = 9.0(1) GPa an apparent kink followed by a slower decreasing trend is evident until reaching a value of 1.832(1) Å at 16.5(2) GPa. It is interesting to note that *d*_nn-c_ and *u* are essentially following a similar trend of pressure dependence over the entire pressure range investigated in this study, implying the prominent role played by the nearest-neighbor bonding along the *c*-axis.

**Figure 5:**
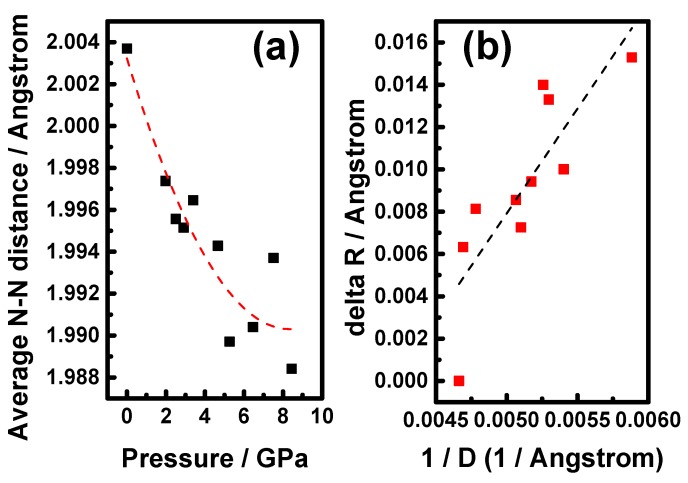
(**a**) Pressure dependence of the N-N distances; and (**b**) the N-N distance contraction versus the inverse of A_3_ZO-NPs size.

Figure 5a shows the reduced average nearest-neighbor (N-N) distance *R* of *d*_nn-c_ and *d*_nn-et_ and average particle size *D* of the B4 phase as a function of the applied pressure. It is evident from Figure 5a that the linear compressibility of the average nearest neighbor distances and average particle sizes are *K_R_* = −[1/R_0_][*d*(R)/*dP*]*_T_* = 1.547 × 10^−3^ GPa^−1^ and *K_D_* = −[1/D_0_][*d*(D)/*dP*]*_T_* = 1.547 × 10^−3^ GPa^−1^, respectively, up to 8.4(1) GPa. Here, R_0_ and D_0_ are the average N-N distance and average particle size at ambient pressure, respectively. Especially, the particle size D is 17.0 nm for 8.4(1) GPa, which is about 20% reduction compared to that of 21.4 nm at ambient pressure. Evidently, increasing of hydrostatic pressure would reduce the cell volume, the average nearest neighbor distances and particle sizes as expected. Figure 5b shows that the contraction of average N-N distance Δ*R* ≡ (*R*_0_ − *R*) of the B4 phase increases linearly with the inverse of average particle size 1/*D*. Apai et al. [61] and Balerna et al. [62] argued from a macroscopic point of view (liquid drop model) and pointed out that Δ*R* should be closely related to the surface stress. Cammarata [63] further indicated that the surface stress reflects the reversible work required to elastically stretch a surface, which is manifested by redistribution of electron density around surface atoms and can be both positive (tensile) or negative (compressive). Accordingly, we can modify the liquid drop model [61,62] such that, under high pressure, the contraction of the N-N distance becomes Δ*R* = −(4/3)*KfR*_0_/*D*, where *f* is the surface stress and *K* is the bulk compressibility (the inverse of the bulk modulus). The slope of the straight line fit in Figure 5b is 9.907(1) Å^2^. The bulk values of *R*_0_ and *K* are 2.004(1) Å and 1/146 GPa^−1^, respectively. These values, in turn, give rise to a surface stress *f* ≈ −54.139(1) J m^−2^ for the present A_3_ZO-NPs. We note that the obtained value is about 78 times more than that of ZnO nanoparticles (≈0.69 J∙m^−2^ [24]) under ambient pressure. It should be able to provide enough of the reversible work to account for the 20% reduction in particle size at high pressure described above.

**Figure 6:**
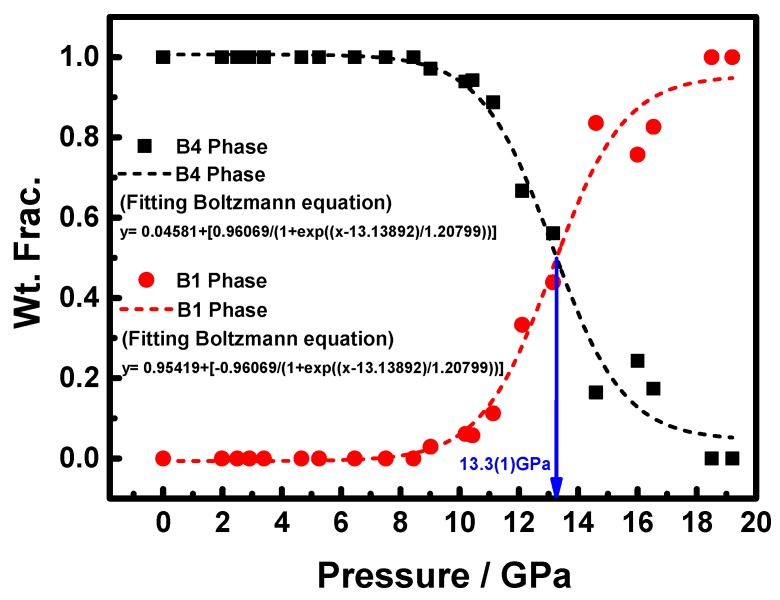
Pressure dependence of the weight fraction (Wt. Frac.) of B4 and B1 phases at 300 K in A_3_ZO-NPs.

Figure 6 shows the weight fraction (Wt. Frac.) as a function of pressure for both B4 and B1 phases. The results indicate that the intermediate pressure range B4 and B1 phases coexist and the amount of increasing B1 corresponds well with that of the decreasing B4 as the pressure is continuously increased. This again indicates that the phase transition is taking place locally rather than globally. The pressure of equal weight fraction value (50:50) of B4 and B1 phases is around 13.3(1) GPa by a sigmoidal fit of Boltzmann function. To date, the effect of local phase transition has remained largely unanswered. Since the pressure of equal weight fraction value is not available for pure bulk ZnO under compression, we can only estimate the pressure of equal weight fraction value of B4 and B1 phases to be around 11.6 GPa from the results reported by Duzynska et al. [27]. From the results, it is suggestive that the effect of doping impurity ions into pure bulk ZnO might be the primary reason of increasing the pressure of equal weight fraction value under compression. To explain why A_3_ZO-NPs have lower phase-transition pressure than that of Zn_0.97_Al_0.03_O bulks, it is heuristic to compare the behaviors observed in ZnO nanocrystals [24,25,26,27]. The results clearly predict that the phase transition pressure, for A_3_ZO-NPs with grain size of 21.4(1) nm, *P*_tr-nano_ (21.4(1) nm) = 12.7(1) GPa, which is very close to the pressure (*P* = 13.3(1) GPa) indicated in Figure 6, at which B4 and B1 phases are having equal weight fraction value. In A_3_ZO-NPs, Al^3+^ and Zn^2+^ are mainly residing in octahedral and tetrahedral sites, respectively, under ambient pressure [17,64]. Jaffe [64] reported that Zn^2+^ has two electrons paired in each of the five 3*d* orbitals, resulting in an algebraic cancellation of increased and decreased energies and leading to a net crystal field stabilization energy (CFSE) Δ_0_ = 0. On the other hand, Al^3+^ favors the octahedral sites, which would result in reduction of interatomic distances and lead to higher lattice energy and increased instability. Our data show that both the slight reduction in unit-cell parameters and structural distortion are evident by introducing dilute Al^3+^ into octahedral sites randomly to substitute for Zn^2+^ residing on the tetrahedral sites, which might account for the reduction of the phase-transition pressure from 12.7(1) for 21.4(1) nm ZnO [24,25,26,27] to 9.0(1) GPa for A_3_ZO-NPs. From Figure 3a, the experimental enthalpy change (Δ*H*) for the reduction of the phase-transition pressure may be estimated as follows [49]: Δ*H* ≈ Δ*P*_tr_ (−Δ*V*) = 3.7 GPa × 3.933 Å^3^ × 6.02 × 10^23^/mole = 8.760(1) KJ∙mole^−1^, which can provide enough internal energy to account for the reduction of the phase-transition pressure.

## 4. Conclusions

In summary, AZO-NPs with the hollowed plate-like and granular microstructures were prepared by chemical precipitation method followed by crystallization at 500 °C. With this method, Al doping concentration up to 11 at % has been achieved without any discernible metal ion clustering or impurity phases. The ADXRD spectra indicate that the as-prepared A_3_ZO-NPs are single-phase B4-structured ZnO at ambient pressure, suggesting that aluminum atoms are completely doped into the ZnO lattice by substituting for zinc atoms. High-pressure ADXRD with pressures up to 19.2(1) GPa revealed that the B4-to-B1 phase transition in this A_3_ZO-NPs has an onset transition pressure of ~9.0(1) GPa. The compressibility of A_3_ZO-NPs is anisotropic with that along the *c*-axis being larger than that along the *a*-axis. The largest change in the *u* value with increasing pressure is found to originate mainly from stretching the nearest-neighbor distance of O to Zn(Al) parallel to the *c*-axis for A_3_ZO-NPs. Comparing with the phase-transition pressure of 12.7(1) for 21.4(1) nm ZnO, the reduction in phase-transition pressure of A_3_ZO-NPs is believed to be mainly due to the fact that Zn^2+^ and Al^3+^ are preferably occupying the tetrahedral and octahedral sites, respectively. The fitting with equation of state for the A_3_ZO-NPs gives a bulk modulus and its first derivative being of 146(7) GPa and 7(2), respectively. An increase in the *u* value with increased pressure indicates that the B4-to-B1 phase transformation in A_3_ZO-NPs is more likely via the hexagonal rather than the tetragonal path. Finally, it is noted that during the decompress process, the B1-strucutre can sustain even when the applied pressure is completely relaxed, suggesting that the pressure-induced phase transition in these A_3_ZO-NPs is irreversible.

## Figures and Tables

**Table 1:** The unit-cell parameters at the ambient pressure of AZO-NPs.

AZO-NPs	*A* (Å)	*C* (Å)	Atomic Volume (Å^3^)
Zn_0.99_Al_0.01_O	3.248(3)	5.202(1)	23.788(2)
Zn_0.97_Al_0.03_O	3.247(1)	5.199(1)	23.737(2)
Zn_0.94_Al_0.06_O	3.246(3)	5.197(2)	23.783(2)
Zn_0.91_Al_0.09_O	3.245(4)	5.195(1)	23.779(2)
Zn_0.89_Al_0.11_O	3.244(3)	5.190(2)	23.776(1)

**Table 2:** The concentration of Al in AZO-NPs measurements by EDS and ICP-AES analyses.

	AZO-NPs	Zn_0.99_Al_0.01_O	Zn_0.97_Al_0.03_O	Zn_0.94_Al_0.06_O	Zn_0.91_Al_0.09_O	Zn_0.89_Al_0.11_O
ICP-AES						
measured concentration of Zn/mg L^−1^		17.05	32.61	25.91	27.63	30.80
measured concentration of Al/mg L^−1^		0.15	0.49	0.73	1.02	1.55
measured mole of Zn/μmole		13.04	24.94	19.81	21.13	23.55
measured mole of Al/μmole		0.27	0.90	1.36	1.89	2.87
atomic concentration/at %		2.03	3.48	6.42	8.21	10.86

**Table 3:** Unit-cell lattice parameters *a*, *c*, *c*/*a*, *u*, the nearest-neighbor distance between O^2−^ and Zn^2+^(Al^3+^) ions measured the *c*-axis (*d*_nn-c_) and the bond length of equilateral triangle (*d*_nn-et_) of A_3_ZO-NPs of B4 phase before 16.5(2) GPa.

Pressure (GPa)	*A* (Å)	*C* (Å)	*c*/*a*	*u*	*d*_nn-c_ (Å)	*d*_nn-et_ (Å)
ambient	3.247(1)	5.199(1)	1.601(1)	0.400(1)	2.067(1)	1.940(1)
2.0(1)	3.238(1)	5.178(1)	1.599(1)	0.402(1)	2.049(1)	1.946(1)
2.5(1)	3.236(1)	5.169(1)	1.597(1)	0.401(1)	2.047(1)	1.944(1)
2.9(1)	3.234(1)	5.159(1)	1.596(1)	0.403(1)	2.050(1)	1.941(1)
3.4(1)	3.233(1)	5.156(1)	1.595(1)	0.404(1)	2.054(1)	1.938(1)
4.6(1)	3.228(2)	5.132(1)	1.591(1)	0.406(1)	2.059(1)	1.930(1)
5.3(1)	3.225(2)	5.130(1)	1.591(2)	0.405(1)	2.047(1)	1.932(1)
6.5(1)	3.219(1)	5.117(1)	1.589(1)	0.407(1)	2.055(1)	1.926(1)
7.5(1)	3.216(1)	5.109(1)	1.588(1)	0.404(1)	2.068(1)	1.919(1)
8.4(1)	3.212(1)	5.102(1)	1.588(2)	0.405(1)	2.058(1)	1.918(2)
9.0(1)	3.207(1)	5.094(1)	1.588(1)	0.407(1)	2.067(1)	1.913(1)
10.2(1)	3.204(1)	5.092(1)	1.589(1)	0.408(1)	2.102(1)	1.901(1)
10.4(1)	3.203(2)	5.091(2)	1.590(1)	0.409(1)	2.121(2)	1.898(1)
11.1(2)	3.201(2)	5.087(2)	1.590(1)	0.414(1)	2.176(1)	1.884(1)
12.1(1)	3.194(1)	5.082(1)	1.591(1)	0.428(1)	2.297(1)	1.860(1)
12.9(2)	3.193(1)	5.082(2)	1.592(1)	0.433(1)	2.393(1)	1.846(1)
14.6(1)	3.192(1)	5.081(1)	1.591(1)	0.443(1)	2.521(1)	1.835(1)
16.0(1)	3.188(1)	5.080(1)	1.594(1)	0.470(1)	2.536(1)	1.832(2)
16.5(2)	3.190(1)	5.079(1)	1.592(1)	0.498(1)	2.590(1)	1.831(1)

## References

[1] Mang A., Reimann K., Rubenacke S. (1995). Band gaps, crystal-field splitting, spin-orbit coupling, and exciton binding energies in ZnO under hydrostatic pressure. Solid State Commun..

[2] Mohanta A., Thareja R.K. (2009). Rayleigh scattering from gaseous phase nanoparticles synthesized by pulsed laser ablation of ZnO. J. Appl. Phys..

[3] Mohanta A., Singh V., Thareja R.K. (2008). Photoluminescence from ZnO nanoparticles in vapor phase. J. Appl. Phys..

[4] Mohanta A., Simmons J.G., Everitt H.O., Shen G., Kim S.M., Kung P. (2014). Effect of pressure and Al doping on structural and optical properties of ZnO nanowires synthesized by chemical vapor deposition. J. Luminescence.

[5] Mohanta A., Thareja R.K. (2014). Photoluminescence characteristics of catalyst free ZnO nanowires. Mater. Res. Express.

[6] Mohanta A., Thareja R.K. (2008). Photoluminescence study of ZnO nanowires grown by thermal evaporation on pulsed laser deposited ZnO buffer layer. J. Appl. Phys..

[7] Huang J.M., Tsai S.Y., Ku C.S., Lin C.M., Chen S.Y., Lee H.Y. (2016). Enhanced electrical properties and field emission characteristics of AZO/ZnO-nanowire core–shell structures. Phys. Chem. Chem. Phys..

[8] Thareja R.K., Saxena H., Narayanan V. (2005). Laser-ablated ZnO for thin films of ZnO and Mg*_x_*Zn_(1−*x*)_O. J. Appl. Phys..

[9] Mohanta A., Thareja R.K. (2008). Photoluminescence study of ZnCdO alloy. J. Appl. Phys..

[10] Mohanta A., Thareja R.K. (2010). Temperature-dependent S-Shaped photoluminescence in ZnCdO alloy. J. Appl. Phys..

[11] Fathollahi V., Amini M.M. (2001). Sol–Gel Preparation of Highly Oriented Gallium-Doped Zinc Oxide Thin Films. Mater. Lett..

[12] Arredondo E.J.L., Maldonado A., Asomoza R., Acosta D.R., Lira M.A.M., Olvera M., de la L. (2005). Indium-Doped ZnO Thin Films Deposited by the Sol-Gel Technique. Thin Solid Films.

[13] Srinivasan G., Rajendra Kumar R.T., Kumar J. (2007). Influence of Al dopant on microstructure and optical properties of ZnO thin films prepared by sol–gel spin coating method. Opt. Mater..

[14] Shirouzu K., Ohkusa T., Hotta M., Enomoto N., Hojo J. (2007). Distribution and Solubility Limit of Al in Al_2_O_3_-Doped ZnO Sintered Body. J. Ceram. Soc. Jpn..

[15] Kadam P., Agashe C., Mahamuni S. (2008). Al-doped ZnO nanocrystals. J. Appl. Phys..

[16] Chen K.J., Fang T.H., Hung F.Y. (2008). The crystallization and physical properties of Al-doped ZnO nanoparticles. J. Appl. Surf. Sci..

[17] Serier H., Gaudon M., Ménétrier M. (2009). Al-doped ZnO powdered materials: Al solubility limit and IR absorption properties. Solid State Sci..

[18] Bazer-Bachi D., Coupard V., Maury S., Rebours B. (2013). Method of Preparing Alcohol Esters from Triglycerides and Alcohols Using Heterogeneous Catalysts Combining at Least a Zn_x_Al_2_O_3+*x*_ Type Solid Solution and ZnO. U.S. Patent.

[19] Chen Z., Zhan G., Lu Z. (2014). Solvothermal synthesis and conductive properties of nanorod-constructed Al-doped ZnO microflowers. J. Mater. Sci.: Mater. Electron.

[20] Suwanboon S., Amornpitoksuk P., Haidoux A., Tedenac J.C. (2008). Structural and optical properties of undoped and aluminium doped zinc oxide nanoparticles via precipitation method at low temperature. J. Alloys Compd..

[21] Kim H., Gilmore C.M., Piqué A., Horwitz J.S., Mattoussi H., Murata H., Kafafi Z.H., Chrisey D.B. (1999). Electrical, optical, and structural properties of indium-tin-oxide thin films for organic light-emitting devices. J. Appl. Phys..

[22] Minami T. (2005). Transparent conducting oxide semiconductors for transparent electrodes. Semicond. Sci. Tech..

[23] Meyer J., Gorrn P., Hamwi S., Johannes H.H., Riedl T., Kowalsky W. (2008). Indium-free transparent organic light emitting diodes with Al doped ZnO electrodes grown by atomic layer and pulsed laser deposition. Appl. Phys. Lett..

[24] Bayarjargal L., Wiehl L., Winkler B. (2013). Influence of grain size, surface energy, and deviatoric stress on the pressure induced phase transition of ZnO and AlN. High Press. Res..

[25] Jiang J.Z., Olsen J.S., Gerward L., Frost D., Rubie D., Peyronneau J. (2000). Structural stability in nanocrystalline ZnO. Europhys. Lett..

[26] Grzanka E., Gierlotka S., Stelmakh S., Palosz B., Strachowski R., Swiderska S.A., Kalisz G., Lojkowski W., Porsch F. (2006). Phase transition in nanocrystalline ZnO. Z. Kristallogr. Suppl..

[27] Duzynska A., Hrubiak R., Drozd V., Teisseyre H., Paszkowicz W., Reszka A., Kaminska A., Saxena S., Fidelus J.D., Grabis J.C. (2012). The structural and optical properties of ZnO bulk and nanocrystals under high pressure. High Pressure Res..

[28] Wang Y., Zhang Y., Chang W.J., Lu G.L., Jiang J.Z., Li Y.C., Liu J., Hu T.D. (2005). Mn effect on wurtzite-to-cubic phase transformation in ZnO. J. Phys. Chem. Solids.

[29] Lin C.M., Lin K.L., Chern Y.K., Hsua C.H., Sheu H.S., Liao Y.F., Suen Y.W., Jian S.R., Juang J.Y. (2014). Pressure-induced structural phase transition in bulk Zn_0.98_Mn_0.02_O by angular dispersive X-ray diffraction. J. Alloys Compd..

[30] Niesz D.E., Bennett R.B., Snyder M.J. (1972). America Ceramic society. Am. Ceram. Soc. Bull..

[31] Mao H.K., Xu J., Bell P.M. (1986). Calibration of the Ruby Pressure Gauge to 800 kbar Under Quasi. J. Geophys. Res..

[32] Angel R.J., Bujak M., Zhao J., Gatta G.D., Jacobsen S.D. (2007). Effective hydrostatic limits of pressure media for high-pressure crystallographic studies. J. Appl. Crystallogr..

[33] Klotz S., Chervin J.C., Munsch P., Marchand G.L. (2009). Hydrostatic limits of 11 pressure transmitting media. J. Phys. D Appl. Phys..

[34] Larson A.C., von Dreele R.B. (2000). General Structure Analysis System (GSAS).

[35] Mote V.D., Purushotham Y., Dole B.N. (2012). Williamson-Hall analysis in estimation of lattice strain in nanometer-sized ZnO particles. J. Theor. Appl. Phys..

[36] Brehm J.U., Winterer M., Hahn H. (2006). Synthesis and local structure of doped nanocrystalline zinc oxides. J. Appl. Phys..

[37] Ku C.S., Huang J.M., Cheng C.Y., Lin C.M., Lee H.Y. (2010). Annealing effect on the optical response and interdiffusion of n-ZnO/p-Si (111) heterojunction grown by atomic layer deposition. Appl. Phys. Lett..

[38] Farbod M., Shoushtari M.Z., Parhoodeh S. (2011). Fabrication and characterization of Zn_1−*x*_Al*_x_*O nanoparticles by DC arc plasma. Phys. B Condens. Matter..

[39] Noh J.H., Han H.S., Lee S., Kim D.H., Park J.H., Park S., Kim J.Y., Jung H.S., Hong K.S. (2010). A newly designed Nb-doped TiO_2_/Al-doped ZnO transparent conducting oxide multilayer for electrochemical photoenergy conversion devices. J. Phys. Chem. C.

[40] Choi Y.J., Gong S.C., Park C.S., Lee H.S., Jang J.G., Chang H.J., Yeom G.Y., Park H.H. (2013). Improved performance of organic light-emitting diodes fabricated on Al-doped ZnO anodes incorporating a homogeneous Al-doped ZnO buffer layer grown by atomic layer deposition. ACS Appl. Mater. Interfaces.

[41] Alberti A., Marco L.D., Pellegrino G., Condorelli G.G., Giannuzzi R., Scarfiello R., Manca M., Spinella C., Gigli G., Magna A.L. (2014). Combined strategy to realize efficient photoelectrodes for low temperature fabrication of dye solar cells. ACS Appl. Mater. Interfaces.

[42] Yu S.C., Spain I.L., Skelton E.F. (1978). Lattice dynamics and hyperfine interactions in ZnO and ZnSe at high external pressures. Solid State Commun..

[43] Karzel H., Potzel W., Köfferlein M., Schiessel W., Sreiner M., Hiller U., Kalvius G.M., Mitchell D.W., Das T.P., Blaha P. (1996). High pressure phase transitions in tetrahedrally coordinated semiconducting compounds. Phys. Rev. B.

[44] Gerward L., Staun Olsen J. (1995). The High-Pressure Phase of Zincite. J. Synchrotron Radiat..

[45] Desgreniers S. (1998). High-density phases of ZnO: Structural and compressive parameters. Phys. Rev. B.

[46] Liu H., Ding Y., Somayazulu M., Qian J., Shu J., Häusermann D., Mao H.K. (2005). Rietveld refinement study of the pressure dependence of the internal structural parameter u in the wurtzite phase of ZnO. Phys. Rev. B.

[47] Sowa H., Ahsbahs H. (2006). High-pressure X-ray investigation of zincite ZnO single crystals using diamond anvils with an improved shape. J. Appl. Crystallogr..

[48] Cai J. (2007). First-principles study of the wurtzite-to-rocksalt phase transition in zinc oxide. J. Phys. Condens. Matter.

[49] Hemley R.J. (1998). Ultrahigh-Pressure Mineralogy: Physics and Chemistry of the Earth’s Deep Interior.

[50] Decremps F., Datchi F., Saitta A.M., Polian A., Pascarelli S., Cicco A.D., Itie J.P., Baudelet F. (2003). Local structure of condensed zinc oxide. Phys. Rev. B.

[51] Dong Z., Zhuravlev K.K., Morin S.A., Li L., Jin S., Song Y. (2012). Pressure-Induced Structural Transformations of ZnO Nanowires Probed by X-ray Diffraction. J. Phys. Chem. C.

[52] Wang L., Liu H., Qian J., Yang W., Zhao Y. (2012). Structural Stability and Compressibility Study for ZnO Nanobelts under High Pressure. J. Phys. Chem. C.

[53] Lin C.M., Lin K.L., Chern Y.K., Lin Y.K., Chuang Y.C., Liao Y.F., Suen Y.W., Jian S.R., Juang J.Y. (2015). Pressure-Induced Phase Transitions in InAs Studied by Angular-Dispersive X-ray Diffraction and Raman Spectroscopy. Sci. Adv. Mater..

[54] Saitta A.M., Decremps F. (2004). Unifying description of the wurtzite-to-rocksalt phase transition in wide-gap semiconductors: The effect of d electrons on the elastic constants. Phys. Rev. B.

[55] Limpijumnong S., Jungthawan S. (2004). First-principles study of the wurtzite-to-rocksalt homogeneous transformation in ZnO: A case of a low-transformation barrier. Phys. Rev. B.

[56] Kisi E., Elcombe M.M. (1989). Parameters for the Wurtzite Structure of ZnS and ZnO using Powder Neutron Diffraction. Acta Crystallogr. Sect. C Cryst. Struct. Commun..

[57] Pauling L. (1932). The nature of the chemical bond. iv. The energy of single bonds and the relative electronegativity of atoms. J. Am. Chem. Soc..

[58] Kumar R.S., Cornelius A.L., Nicol M.F. (2007). Structure of nanocrystalline ZnO up to 85 GPa. Curr. Appl. Phys..

[59] Hou D.B., Ma Y.Z., Gao C.X., Chaudhuri J.R., Lee G., Yang H.B. (2009). Compression of a crystalline ZnO nanotube: An experimental exploration of the B4 to B1 transition mechanism. J. Appl. Phys..

[60] Özgür Ü., Alivov Y.I., Liu C., Teke A., Reshchikov M.A., Doğan S., Avrutin V., Cho S.J., Morkoç H. (2005). A comprehensive review of ZnO materials and devices. J. Appl. Phys..

[61] Apai G., Hamilton J., Stohr J., Thompson A. (1979). Extended X-ray—Absorption Fine Structure of Small Cu and Ni Clusters: Binding-Energy and Bond-Length Changes with Cluster Size. Phys. Rev. Lett..

[62] Balerna A., Bernieri E., Picozzi P., Reale A., Santucci S., Burattini E., Mobilio S. (1985). Extended X-ray-absorption fine-structure and near-edge-structure studies on evaporated small clusters of Au. Phys. Rev. B.

[63] Cammarata R.C. (1994). Surface and Interface Stress Effects in Thin Films. Prog. Surf. Sci..

[64] Jaffe H.W. (1988). Introduction to Crystal Chemistry.

